# Meal-specific dietary patterns and biomarkers of insulin resistance in a sample of Iranian adults: a cross-sectional study

**DOI:** 10.1038/s41598-023-34235-3

**Published:** 2023-05-08

**Authors:** Azadeh Lesani, Ahmad Jayedi, Mehrdad Karimi, Kurosh Djafarian, Bahareh Barkhidarian, Zahra Akbarzade, Sakineh Shab-Bidar

**Affiliations:** 1 Department of Community Nutrition, School of Nutritional Sciences and Dietetics, Tehran University of Medical Sciences, Tehran, Iran; 2 Khoy University of Medical Sciences, Khoy, West Azerbaijan Iran; 3 Department of Clinical Nutrition, School of Nutritional Sciences and Dietetics, Tehran University of Medical Sciences, Tehran, Iran

**Keywords:** Diseases, Endocrinology, Health care, Risk factors

## Abstract

Current research emphasizes the habitual dietary pattern without differentiating eating occasions. We aimed to assess meal-specific dietary patterns and insulin resistance indicators. This cross-sectional study was conducted on 825 Iranian adults. Dietary data were recorded by three 24-h dietary recalls. Dietary patterns were identified using principal component analysis (PCA) on main meals and an afternoon snack. Anthropometric measurements, blood pressure, and laboratory investigation, fasting plasma glucose (FPG), triglyceride, insulin, c-reactive protein (CRP) were done. Homeostatic model assessment for insulin resistance and sensitivity (HOMA-IR and HOMA-IS), Triglycerides and glucose (TyG-index), and Lipid accommodation product index were calculated. We used multivariate analysis of variance (MANOVA) analysis. Two major dietary patterns at the main meals and the afternoon were identified. Higher adherence to “Bread, vegetable, and cheese” dietary pattern at breakfast was related to lower FPG, and “Oil, egg, and cereals” showed a direct association with body mass index, FPG, and TyG-index at breakfast. The “Western” pattern in lunch and dinner directly related to waist circumference (WC) and body mass index, however, it was inversely related to HOMA-IS. This pattern at dinner was related to higher CRP. Higher adherence to “bread, cereals, and oil” pattern at afternoon snack was associated with a lower WC. These results indicated that unhealthy meal-specific dietary patterns are associated with a greater chance of obesity and insulin resistance risk. However, “Bread, vegetable, and cheese” dietary pattern at breakfast was related to lower FPG, and “bread, cereals, and oil” pattern in the afternoon was associated with lower WC.

## Introduction

Insulin resistance (IR) is one of the most important topics in nowadays medicine. IR generally refers to decreased insulin sensitivity in the human tissues^1^. Abnormal structure of insulin molecule, deteriorated signaling pathways, and declined function of insulin receptors may play a key role in the development of IR^2^. Accumulative evidence suggests that IR is an underlying cause of several cardiometabolic abnormalities such as dyslipidemia, impaired glucose tolerance, and hyperinsulinemia and thus, is associated with a higher risk of developing type 2 diabetes^3^, cardiovascular disease (CVD)^4^, and site-specific cancer risk^5^. There is also a bidirectional association between obesity and IR, in a way that obesity could lead to the incidence of IR and in return, IR could lead to the development of overweight and obesity^6^.

Given the global prevalence of non-communicable chronic disease and considering the underlying role of IR in developing chronic diseases, there is a pressing need to investigate modifiable risk factors implicated in developing IR. Studies have suggested a potential link between dietary habits and IR^7^. Evidence suggested that intake of some plant-based food groups such as whole grains^8^, vegetables^9^, and nuts^10^ may improve insulin resistance and in contrast, the consumption of red meat^11^ and soft drink^12^ may be associated with abnormal insulin sensitivity. Evidence from epidemiologic studies also suggest a potential link between healthy and unhealthy dietary patterns and IR^13–15^.

However, most of the studies addressing the association of dietary patterns and IR have focused on habitual dietary patterns^13–15^. Indeed, limited evidence is available about the association between meal-specific dietary patterns with IR and other cardiometabolic abnormalities^16,17^. Foods are consumed on different eating occasions across the day named meals. Recent studies have suggested that meal-specific dietary habits such as meal timing and frequency may be associated with multiple health outcomes^18–22^ Chrononutrition is an emerging field in nutrition research that focuses on the potential interaction between dietary habits and circadian rhythm and investigates how well meal timing and frequency and quality of foods consumed at each meal are associated with health consequences^18,23^.

Studies have indicated that breakfast skipping^24–26^, energy contribution by meals^27,28^ and the number of eating occasions across the day^29^ could have an influence on health outcomes. The 2017 American Heart Association scientific statement suggested that meal-specific eating styles such as meal timing and frequency may be associated with cardiometabolic health and suggested focusing on such meal-specific properties to achieve a healthier lifestyle and improved risk factor management^30^. However, limited evidence is available about the association between meal-specific eating styles and cardiometabolic abnormalities. To our knowledge, no study has examined the potential association between meal-specific data-driven dietary patterns and biomarkers of IR in Iran. To address this gap, we performed a cross-sectional study to investigate whether meal-specific data-driven dietary patterns, identified by a data-reduction statistical approach, are associated with biomarkers of IR among Iranian adults.

## Subjects and methods

### Study design and participants

This cross-sectional study was conducted in apparently healthy men and women from Iran who attended health care centers of Tehran from February 2019 to August 2019. A sample size of 820 participants was calculated based on the following the formula n = ((z_α_ + z_β_)/(0.5 × In [(1 + r)/(1-r)]))^2^ + 3^31^, where in r was the correlation between whole wheat bread and Homeostatic Model Assessment for Insulin Resistance (HOMA-IR) (r = 0.098)^32^, and an α level of 0.05 and 1–β of 80%. Participants were recruited using a two-stage cluster sampling method within 25 healthcare centers across five different geographic areas of Tehran. A convenient sampling method was used to select the study participants from each health center, using the proportion-to-size approach. The inclusion criteria were having 18–59 years old and a body mass index (BMI) of 18.5–39.9 kg/m^2^. The exclusion criteria were pregnancy or lactation, and having a chronic disease.

### Ethical approval

The study was ethically approved by the Ethics Committee of Tehran University of Medical Sciences (IR.TUMS.Medicine.REC.1399.295). The purpose of the study was explained to the participants, and all participants were given written informed consent precede to enter the study. The methods were conducted in accordance with the relevant Declaration of Helsinki guidelines and regulations.

### Dietary intake assessment and meal timing

Dietary data were obtained based on three 24-h dietary recalls on non-consecutive days within the week. We conducted all recalls by trained dietitians during a private interview. The first 24-h dietary recall was recorded on the first visit in the health care center. The following recalls were collected via telephone on random days. A total of 2459 recalls were recorded. Subjects reported the following types of eating occasions in which food was consumed: breakfast, lunch, dinner, or snacks. The definition of main meals and afternoon snake according to the time of food intake was explained in a prior article^23^.

Daily intakes of all food items, derived from three 24-h dietary recalls, were converted into grams per day by using household measures^33^. Intake of food groups was adjuster for energy intake by using the residual method^34^. We used the Nutritionist IV software (First Databank, San Bruno, CA, USA), modified for Iranian foods, to obtain the values of energy and nutrients intake per day. A total of 420 food items were derived from 24-h dietary recalls and were classified into 26 food groups (Supplementary Table 1) based on the similarity of nutrient content in each food item and a literature search^22,35–37^. Every food group consumed at meals was used to extract meal-specific dietary patterns.

### Data collection

Data were collected from each person by a face-to-face interview. Sociodemographic characteristics were collected by using pre-specified data extraction forms and included age, gender, marriage status (single, married, divorced), income (monthly income), smoking status (not smoking, ex-smoking, current smoking), education level (illiterate, under diploma and diploma, educated), occupation status (employed, unemployed, retired), supplement intake (yes or no) and living status (live alone or live with someone).

### Physical activity

Physical activity was measured by the short form of the validated International Physical Activity Questionnaire (IPAQ)^38^. Participants reported the time spent walking or doing moderate- and/or vigorous-intensity activities within the previous seven days. The overall physical activity level was measured in the form of metabolic equivalent minutes per week (MET-minutes/week). MET scores were then categorized into three levels: point score < 600 MET-min/week as low physical activity, point score 600–3000 MET-min/week as moderate physical activity, and point score > 3000 MET-min/week as high physical activity^39^.

### Assessment of blood pressure

Blood pressure was measured on the right hand by a digital barometer (BC 08, Beurer, Germany) after at least 10–15 min of rest and sitting. Blood pressure was measured twice for every person, and the average of the two measurements was reported for each person.

### Anthropometric measurements

Weight was measured using a Seca weighing scale (Seca and Co. KG; 22 089 Hamburg, Germany; Model: 874 1321009; designed in Germany; made in China) with light clothing (without a coat and raincoat). A wall stadiometer board with a sensitivity of 0.1 cm was used to measure standing height, without shoes. BMI was calculated as weight (WT) in kilograms divided by height (HT) in meters squared (BMI: WT/HT^2^). Waist circumference (WC) was measured using a non-stretchable fiberglass measuring tape at the midpoint between the lower border of the rib cage and the iliac crest. Waist-hip ratio (WHR) was calculated for each person by dividing WC by hip circumference.

### Laboratory investigations

All participants donated ten ml of blood between the hours 7–10 am in a fasted status. Following this, blood samples were collected in acid-washed test tubes without anticoagulants. Then, it was being stored at room temperature for thirty minutes and clot formation, blood samples were centrifuged at 1500 g for twenty minutes. Serums were stored at – 80 °C until future testing. Fasting plasma glucose (FPG) was assayed by the enzymatic (glucose oxidase) colorimetric method using a commercial kit (Pars Azmun, Iran, Pars Azmun Inc.). Serum total (TC) and high-density lipoprotein cholesterol (HDL-C) were measured using a cholesterol oxidase phenol aminoantipyrine method, and serum triglyceride (TG) was measured using a glycerol-3 phosphate oxidase phenol aminoantipyrine enzymatic method. Serum low-density lipoprotein cholesterol (LDL-C) was calculated using the Friedewald formula^40^. Serum insulin concentration was measured using the commercial kits (AccuBind Insulin ELIZA, USA, Monobind Inc.) and enzyme-linked immunosorbent assay (ELISA) method. Serum uric acid was measured by the calorimetry method using commercial kits (Bionic, Iran, Bionic Inc.) and biolysis 24. Serum C-reactive protein (CRP) was measured by a commercial kit (CRP LX (300 T) cobass c intergra, Germany, Roche Inc.) by the immunoturbidimetric method.

### Definition of insulin resistant indicators

Triglycerides and glucose (TyG) index is a marker of insulin resistance^41^, which predicts the development of metabolic disorders and CVD^42^. TyG index was calculated based on the following formula: TyG Index: $\frac{\mathrm{In }(\mathrm{Fasting triglycerides }\left(\mathrm{TG}\right) \left[\frac{\mathrm{mg}}{\mathrm{dL}}\right]*\mathrm{ Glucose }\left(\mathrm{ FPGPG}\right)\left[\frac{\mathrm{mg}}{\mathrm{dL}}\right]) }{2}$.

Lipid Accommodation Product (LAP) index, as a marker of CVD, is a simple indicator of high lipid accumulation in adults^43^, and has greater sensitivity and specificity than waist measures to show insulin resistance^44^. Based on values of WC and fasting TG, the LAP score was calculated using the following formula. Men: (WC _(Cm)_—65) * TG $\left[\frac{\mathrm{mmol}}{\mathrm{L}}\right]$ and Women: (WC _(Cm)_ -58)* TG $\left[\frac{\mathrm{mmol}}{\mathrm{L}}\right]$.

HOMA is a measure of insulin resistance (HOMA-IR) and β-cell function among the diabetic and non-diabetic populations^45^. HOMA of $\beta$-cell function (HOMA-IS) is thought to be a good measure of $\beta$-cell function. High HOMA-IR and low HOMA-IS values were associated with glucose intolerance and subsequent risk of type 2 diabetes^46,47^. HOMA-IR: $\frac{\mathrm{Fasting insulin }\left(\frac{\mathrm{\mu IU}}{\mathrm{mL}}\right)*\mathrm{ FPG }(\frac{\mathrm{mg}}{\mathrm{dL}})}{405}$ and HOMA-IS: $\frac{20*\mathrm{Fasting insulin }\left(\frac{\mathrm{\mu IU}}{\mathrm{mL}}\right)}{\mathrm{FPG }\left(\frac{\mathrm{mg}}{\mathrm{dL}}\right)-3.5}$.

### Statistical analyses

Dietary patterns at meals level (breakfast, lunch, afternoon, and dinner) were determined by principal component analysis (PCA). PCA is a data reduction statistical method that is frequently used to perform dietary pattern analysis and explore posteriori-defined eating patterns in nutrition epidemiologic research^48^. PCA extracts common patterns according to the correlation matrix of food intake^49^. The Kaiser–Meyer–Olkin (KMO) test was used to measure sampling adequacy and Bartlett’s test of sphericity was used to investigate the adequacy of test items and sample size for factor analysis. KMO values > 0.50 was considered as an adequate sample size^50^. The factor loading indicates the correlation between food groups and food patterns and varies from ($-$ 1 to + 1). A positive loading score indicates a positive association with the factor, whereas a negative loading score indicates an inverse association with the factor. Larger positive or negative factor loadings for foods indicate which food groups are important in that component (dietary pattern). The factor loading with magnitude < − 0·2 or > 0.2 were indicated in the tables for simplicity^51^. The number of key dietary patterns to retain was determined based on scree plot analysis (factors with eigenvalues > 1.5) and the interpretability of the identified patterns. Adherence to the meal-based dietary patterns (breakfast, lunch, afternoon, and dinner) was determined based on pattern scores and was categorized into tertiles. Basal Metabolic Rate (BMR) was calculated using standard equations based on weight, age, and sex. Then, the BMR: EI (Basal Metabolic Rate to Energy Intake) is used to assess the validity of the reported amount of energy. Under-reporting of energy intake as BMR: EI < 1.35 and over-reporting as BMR: EI ≥ 2.40 was defined^52^.

Kolmogorov–Smirnov test was used to determine the normal distribution of the data. If the data were not normal, a logarithmic transform was used to normalize them; otherwise, non-parametric tests were used to analyze the data. Demographic, lifestyle characteristics, and health status of the study participants were compared between either sex by using χ^2^ for categorical variables and a t-test for continuous variables. To compare mean and variations of dependent variables across tertiles of meal-specific dietary patterns, we used multivariate analysis of variance (MANOVA) analysis in crude model and after controlling for confounders including age, sex, physical activity, smoking, marital status, income, supplementation, and education. Statistical analyses were conducted using SPSS version 22.0, and P-values < 0.05 were considered statistically significant.

### Ethics approval and consent to participate

The study was ethically approved by the Ethics Committee of Tehran University of Medical Sciences (IR.TUMS.Medicine.REC.1399.295). Informed consent was obtained from all subjects involved in the study.

## Results

The present cross-sectional study was conducted on 850 adults. To avoid misreporting, we excluded 25 participants due to the following reasons: two participants due to underreporting of energy intake (BMR: EI < 1.35), and the other 23 participants due to over-reporting of their energy intake (BMR: EI > 2.40). Finally, 825 participants including 140 men (19.96%) and 685 women (80.04%) with an age range of 20–59 years old and a mean (SD) age of 42.17 (10.5) years, were analyzed. The mean (SD) BMI was 27.1 (4.49) kg/m^2^, and the average calorie intake was 1681 (374) kcal/d. Of 825 participants, 50 participants had two 24-h dietary recalls and the other 775 participants had three 24-h dietary recalls. General characteristics of the study participants are presented in Table 1.

**Table 1:** Baseline lifestyle, sociodemographic and dietary characteristic of the population sample^1^.

Characteristics	
Number (%)	825(100)
Female (%)	685 (80.04)
Age_(Yr)_	42.20 ± 10.60
Physical activity level_(MET.Minutes.WK)_	
Low	430 (52.12)
Moderate	315 (36.96)
High	80 (8.80)
Education	
Illiterate	56 (6.7)
Under diploma and diploma	472 (57.12)
Educated	297 (36.01)
Smoking status	
Not smoking	782 (94.78)
Ex smoking	14 (1.69)
Smoking	29 (3.53)
Supplement intake (yes)	201 (24.3)
Energy intake_(Kcal.Day)_	1681.68 ± 374
Energy intake_(Kcal. Meal_^2^_)_	
Breakfast	418.32 ± 151.54
Lunch	535.86 ± 179.33
Afternoon	170.03 ± 121.21
Dinner	508.10 ± 196.3
SBP (mmHg)	118.25 ± 14.35
DBP (mmHg)	78.3 ± 9.3
WC (cm)	89.09 ± 11.6
WHR	0.86 ± 0.07
BMI _(Kg.m_^2^_)_	27.3 ± 4.5
LAP index ( cm.mmol	49.9 ± 33.9
FPS (mg.dl)	105.3 ± 21.1
TG (mg.dl)	144.5 ± 72.09
$\frac{HDL-C}{LDL-C}$	2.49 ± 0.81
$\frac{TC}{HDL-C}$	4.03 ± 1.05
Uric acid (mg.dl)	4.6 ± 1.3
Insulin serum µU.ml	13.89 ± 12.53
HOMA-IR	3.68 ± 2.9
HOMA-IS	2.66 ± 2.12
CRP	0.23 ± 0.2
TyG- index cm,mg.dl	4.8 ± 2.8

*BMI* body mass index, *SBP* systolic blood pressure, *DBP* diastolic blood pressure, *WC* waist circumference, *WHR* waist hip ratio, *FPG* fasting plasma glucose, *TG* triglyceride, $\frac{HDL-C}{LDL-C}$ ratio high-density lipoprotein to low-density lipoprotein, $\frac{TC}{HDL-C}$ ratio total cholesterol to high-density lipoprotein, *LAP* lipid accumulation product, *HOMA-IR* homeostatic model assessment for insulin resistance, *HOMA-IS* homeostatic model assessment for insulin sensitivity, *CRP* C- reactive protein, *TyG* triglyceride-glucose.

^1^Values are mean ± SD otherwise it is indicated.

PCA identified two principal components (dietary patterns) at each meal (Table 2). Breakfast, lunch, afternoon, and dinner dietary patterns explained 26.97%, 24.97%, 26.34%, and 23.64% of the total variation of diet, respectively. (Supplement Figs. 1–5). The KMO index was 0.54 for breakfast, afternoon and dinner dietary patterns, and 0.57 for lunch dietary patterns. Also, Bartlett’s test was significant (P < 0.001) for all meals.

**Table 2:** Factor loading^2^ on the meal levels and day.

Food groups	Factor loading in meals and day level							
	Breakfast		Lunch		Afternoon		Dinner	
	OEC.DP	BCV.DP	Western, DP	ODEP.DP	BCO.DP	Dessert, soft drink and tea	Western.DP	COPL.DP
Breads		0.391	0.661		0.870		0.439	− 0.321
Cereals and grains	0.321	− 0.544		− 0.512	0.823			0.619
Fresh and dried fruits								
Vegetables		0.356		0.243				0.209
Potato	0.285			0.448		0.221		
Red meat and organ meat				− 0.453				
Poultry		0.424	− 0.277					0.287
Fish								
Processed meat			0.526				0.626	
Broth				0.249	0.297			0.249
Egg	0.683			0.548	0.327			− 0.301
Legume	0.212							0.298
Nut								
Cheese	− 0.662	0.226						
Milk & dairy products		− 0.521	− 0.212	0.521				
Liquid vegetable oils	0.819			0.712	0.620		0.478	0.512
Butter		− 0.286		− 0.245	0.441	0.361		− 0.365
Pickle				0.334				0.234
Salty snacks						0.654		
Sugar and sweets			0.818	− 0.338		0.701	0.838	− 0.338
Industrial beverages and juices			0.844			0.378	0.839	
Tea and herbal tea		0.287						
Coffee								
Sauces							0.436	
Condiments								
Variance of intake explained	15.45	11.42	9.76	15.21	15.33	11.01	14.21	9.46

*OEC.DP* Oil, egg and cereals dietary pattern; *BVC.DP* Bread, vegetable and cheese dietary pattern; *Western.DP* Western dietary pattern; *ODEP.DP* Oil, dairy, egg and potato; *BCO.DP* bread, cereal and oil. dietary pattern; *COPL.DP* cereals, oil, poultry and legume, *COV.DP* cereals, oil and vegetable.dietary pattern, *T* tertiles.

^1^Meal-spesific dietary pattern derived from Principle Component Analysis (PCA).

^2^factor loading is shown while absolute values ≥ 0.3.

Table 2 presents characteristics of the dietary patterns identified at each meal. In breakfast, the pattern labeled “Oil, egg and cereals” was characterized by a high intake of liquid vegetable oils, egg, cereals and grains, legumes, potato, and low intake of milk and dairy products. The second pattern at breakfast was labelled “Bread, vegetable and cheese” and was characterized by high intake in bread, vegetables, cheese, and tea and herbal tea and low intake in butter, milk and dairy products, poultry and cereals and grains. At lunch, the pattern labeled “Western” was featured by bread, industrial beverages and juices, sugar and sweets, processed meat, liquid vegetable oils and low intake in poultry, milk and dairy products. The second pattern at lunch meal, labeled “Oil, dairy, potato and egg”, was characterized by high intake of liquid vegetable oils, milk and dairy products, potato, egg, vegetables, pickle, poultry and broth and low intake in sugar and sweets, butter and red meat and organ meats. In the afternoon, the pattern labelled “bread, cereals and oils” was identified by high intake of bread, cereals and grains, liquid vegetable oils, egg, legume, potato and butter. The second pattern in afternoon was labeled “dessert, soft drink and tee” and was characterized by a high intake in sugar and sweets, industrial beverages and juices, potato, tea and herbal tea. At dinner, the pattern labeled “Western” was featured by bread, industrial beverages and juices, sugar and sweets, processed meat, liquid vegetable oils and sauces. The second pattern labeled “Cereals, oil, poultry, and legume” pattern was characterized by a high intake of cereals and grains, liquid vegetable oils, vegetables, tea and herbal tea, poultry, pickles, and legumes and low intake of bread, egg, and, industrial beverages and juices.

We used MANOVA test to assess the association between meal-specific dietary patterns and biomarkers of obesity, FPG, insulin resistance and inflammation at each meal. Greater adherence to the “bread, vegetables, and cheese” dietary pattern was associated with a lower FPG concentration in crude model (P value = 0.02) and adjusted model (P value = 0.04), in contrast, greater adherence to the “oil, egg, and cereals” dietary pattern was accompanied by a higher BMI in adjusted model (P value = 0.01), a higher FPG concentration in crude model (P value = 0.04) and in adjusted model (P value = 0.01), and a higher TyG-index crude model (P value = 0.007) and adjusted model (P value = 0.004) in adjusted model Table 3. At lunch meal, greater adherence to the “Western” dietary pattern was associated with higher WC (P value = 0.04) and BMI values (P value = 0.04) and a lower HOMA-IS (P value = 0.049) in adjusted model (Table 4).

**Table 3:** Breakfast dietary pattern and cardiometabolic risk factor.

	Breakfast									
	BVC.DP			*P crude	*P adjusted	OEC.DP			*P crude	*P adjusted
	T_1_	T_2_	T_3_			T_1_	T_2_	T_3_		
N	273	279	273			277	274	277		
WC_(cm)_	88.6 ± 12.7	88.8 ± 11.6	89.1 ± 10.6	0.67	0.87	87.9 ± 10.8	88.8 ± 12.4	89.8 ± 12.1	0.92	0.55
BMI_(Kg.m_^2^_)_	27.3 ± 4.2	27.1 ± 4.5	27.1 ± 4.02	0.74	0.86	26.7 ± 4.04	27.1 ± 4.4	27.4 ± 4.6	0.05	0.04*
LAP index	53.3 ± 37.2	48.3 ± 32.5	47.3 ± 36.1	0.46	0.23	44.3 ± 26.3	52.3 ± 28.2	52.3 ± 29.3	0.05	0.21
FPG_(mg.dl)_	107.4 ± 20.1	104.3 ± 19.8	102.9 ± 21.4	0.02*	0.04*	105.3 ± 21.1	105.3 ± 20.5	106.3 ± 20.1	0.04*	0.01*
Uric acid_(mg.dl)_	4.7 ± 1.4	4.6 ± 1.3	4.6 ± 1.2	0.17	0.37	4.5 ± 1.2	4.7 ± 1.3	4.7 ± 1.4	0.13	0.21
Insulin serum_(µU.ml)_	13.8 ± 13.1	13.8 ± 12.8	13.9 ± 11.8	0.99	0.37	13.4 ± 12.8	13.07 ± 12.1	14.3 ± 11.5	0.71	0.77
HOMA-IR	3.6 ± 3.3	3.2 ± 3.3	3.7 ± 3.6	0.65	0.21	3.5 ± 4.3	3.7 ± 3.3	3.8 ± 3.3	0.72	0.59
HOMA-IS	2.61 ± 2.31	2.71 ± 2.23	2.66 ± 1.93	0.99	0.81	2.71 ± 2.36	2.51 ± 1.91	2.63 ± 2.21	0.50	0.21
CRP(µg.dl)	0.24 ± 0.2	0.22 ± 0.19	0.21 ± 0.2	0.86	0.23	0.21 ± 0.19	0.24 ± 0.21	0.23 ± 0.2	0.45	0.98
TyG- index	5.4 ± 2.4	4.9 ± 2.4	4.9 ± 2.4	0.56	0.25	4.4 ± 2.3	4.9 ± 3.5	5.9 ± 2.6	0.007*	0.004*

*OEC.DP* Oil, egg and cereals dietary pattern, *BVC.DP* Bread, vegetable and cheese dietary pattern, *T* tertiles, *BMI* body mass index, *WC* waist circumference, *FPG* fasting plasma glucose, *LAP* lipid accumulation product, *HOMA-IR* homeostatic model assessment for insulin resistance, *HOMA-IS* homeostatic model assessment for insulin sensitivity, *CRP* C- Reactive protein, *TyG* triglyceride-glucose.

Values are mean ± SD.

MANOVA, analysis of covariance used in crude and adjusted model, it adjusted for age, sex, education, physical activity, smoking, income and body mass index.

**Table 4:** Lunch dietary pattern and cardiometabolic risk factors.

	Lunch									
	W.DP			*P crude	*P adjusted	ODEP.DP			*P crude	*P adjusted
	T_1_	T_2_	T_3_			T_1_	T_2_	T_3_		
Number	279	272	274			270	279	276		
WC_(cm)_	87.01 ± 10.9	87.6 ± 10.8	88.8 ± 13.9	0.05	0.04*	87.6 ± 11.09	89.7 ± 11.6	89.1 ± 12.6	0.29	0.20
BMI_(Kg.m_^2^_)_	26.2 ± 4.09	27.3 ± 4.09	27.7 ± 4.9	0.05	0.04*	26.9 ± 4.04	27.1 ± 4.4	27.4 ± 4.7	0.12	0.09
LAP index	49.3 ± 26.2	50.3 ± 28.2	50.3 ± 29.2	0.79	0.78	44.3 ± 26.2	52.3 ± 28.3	52.3 ± 29.2	0.75	0.68
FPG_(mg.dl)_	104.3 ± 20.2	105.3 ± 22.9	105.3 ± 23.2	0.97	0.99	102.4 ± 22.9	103.3 ± 20.6	104.2 ± 20.4	0.71	0.73
Uric acid_(mg.dl)_	4.7 ± 1.3	4.7 ± 1.3	4.6 ± 1.4	0.81	0.85	4.7 ± 1.3	4.6 ± 1.2	4.7 ± 1.4	0.35	0.31
Insulin serum_(µU.ml)_	12.4 ± 10.2	14.4 ± 11.9	14.7 ± 13.7	0.11	0.08	13.8 ± 13.1	14.4 ± 13.7	13.4 ± 10.1	0.62	0.28
HOMA-IR	3.5 ± 4.3	3.7 ± 4.3	3.8 ± 4.3	0.64	0.59	3.6 ± 3.3	3.2 ± 3.3	3.7 ± 4.06	0.55	0.27
HOMA-IS	2.71 ± 2.34	2.57 ± 2.20	2.5 ± 2.12	0.06	0.049*	2.61 ± 2.10	2.72 ± 2.10	2.61 ± 2.24	0.78	0.65
CRP (µg.dl)	10.19 ± 0.18	0.21 ± 0.21	0.21 ± 0.2	0.23	0.14	0.21 ± 0.18	0.24 ± 0.2	0.21 ± 0.2	0.32	0.88
TyG- index	4.4 ± 2.2	4.9 ± 3.5	5.9 ± 2.6	0.29	0.18	5.4 ± 2.4	4.9 ± 2.4	4.9 ± 2.4	0.47	0.42

*W.DP* Western.dietary pattern, *ODEP.DP* Oil, dairy, egg and potato. dietary pattern, *T* tertiles. *BMI* body mass index, *WC* waist circumference, *FPG* fasting plasma glucose, *LAP* lipid accumulation product, *HOMA-IR* homeostatic model assessment for insulin resistance, *HOMA-IS* homeostatic model assessment for insulin sensitivity, *CRP* C- reactive protein, *TyG* triglyceride-glucose.

Values are mean ± SD.

MANOVA analysis of covariance used in crude and adjusted model, and it adjusted for age, sex, education, physical activity, smoking, income and body mass index.

At afternoon meal, a greater adherence to the “bread, cereals, and oil” dietary pattern was associated with a lower WC (P value = 0.02) in adjusted model (Table 5). We also found significant associations between adherence to the Western dietary pattern at dinner meal and some CVD risk factors, in ways that greater adherence to the Western dietary pattern was associated with higher WC (P value = 0.04), BMI (P value = 0.04), and serum CRP concentrations (P value = 0.04) and in contrast, was associated to a lower HOMA-IS value (P value = 0.04) in adjusted model (Table 6).

**Table 5:** Afternoon dietary pattern and cardiometabolic risk factors.

	Afternoon									
	BCO.DP			*P crude	*P adjusted	Dessert, soft drink and tee.DP			*P crude	*P adjusted
	T_1_	T_2_	T_3_			T_1_	T_2_	T_3_		
N	277	274	272			273	277	273		
WC_(cm)_	90.1 ± 10.4	88.6 ± 11.5	88.3 ± 12.3	0.06	0.02*	87.2 ± 11.7	87.6 ± 12.04	87.8 ± 12.9	0.92	0.95
BMI_(Kg.m_^2^_)_	27.2 ± 4.1	27.3 ± 4.4	26.7 ± 4.3	0.25	0.29	26.2 ± 4.09	27.3 ± 4.3	27.7 ± 4.9	0.63	0.71
LAP index	51.3 ± 26.5	50.3 ± 28.6	48.3 ± 27.2	0.70	0.55	48.3 ± 27.1	49.3 ± 28.09	52.3 ± 28.02	0.14	0.08
FPG _(mg.dl)_	105.3 ± 18.2	105.3 ± 22.9	104.3 ± 23.2	0.71	0.57	103.3 ± 20.1	105.3 ± 21.05	106.3 ± 22.5	0.18	0.09
Uric acid _(mg.dl)_	4.6 ± 1.2	4.7 ± 1.2	4.6 ± 1.4	0.76	0.63	4.6 ± 1.2	4.7 ± 1.3	4.7 ± 1.4	0.73	0.68
Insulin serum_(µU.ml)_	13.4 ± 11.4	14.4 ± 11.5	14.7 ± 11.6	0.91	0.71	13.4 ± 11.2	13.4 ± 11.9	14.7 ± 12.7	0.96	0.84
HOMA-IR	3.5 ± 3.3	3.7 ± 3.3	3.8 ± 3.3	0.96	0.63	3.6 ± 3.3	3.6 ± 3.2	3.6 ± 3.5	0.94	0.77
HOMA-IS	2.60 ± 2.26	2.72 ± 2.16	2.64 ± 2.25	0.91	0.9	2.63 ± 2.14	2.72 ± 2.24	2.6 1 ± 2.12	0.62	0.81
CRP (µg.dl)	0.18 ± 0.18	0.22 ± 0.2	0.22 ± 0.21	0.27	0.31	0.19 ± 0.18	0.2 ± 0.19	0.2 ± 0.19	0.65	0.82
TyG- index	4.7 ± 2.2	4.83 ± 3.5	5.01 ± 2.6	0.81	0.43	4.6 ± 2.6	4.9 ± 3.5	5.9 ± 2.8	0.47	0.69

*BCO.DP* Bread, cereals and oil dietary pattern, *OEC.DP* Oil, egg and cereals dietary pattern, *T* tertiles, *BMI* body mass index, *WC* waist circumference, *FPG* fasting plasma glucose, *HOMA-IR* homeostatic model assessment for insulin resistance, *HOMA-IS* homeostatic model assessment for insulin sensitivity, *CRP* C- reactive protein, *TyG* triglyceride-glucose.

Values are mean ± SD.

MANOVA, analysis of covariance used in crude and adjusted model, and it adjusted for age, sex, education, physical activity, smoking, income and body mass index.

**Table 6:** Dinner dietary pattern and cardiometabolic risk factors.

	Dinner									
	W.DP			*P crude	*P adjusted	COPL.DP			*P crude	*P adjusted
	T1	T2	T3			T_1_	T_2_	T_3_		
N	271	281	282			278	273	274		
WC _(cm)_	87.1 ± 10.9	88.6 ± 11.8	90.3 ± 12.9	0.09	0.04*	87.6 ± 11.09	87.7 ± 11.6	86.1 ± 12.6	0.32	0.20
BMI_(Kg.m_^2^_)_	26.2 ± 4.2	27.3 ± 4.4	27.7 ± 4.92	0.10	0.04*	27.3 ± 4.2	27.2 ± 4.4	26.7 ± 4.4	0.09	0.07
LAP index	51.3 ± 26.5	50.3 ± 28.6	48.3 ± 27.3	0.82	0.78	49.3 ± 26.5	50.3 ± 28.6	48.3 ± 26.2	0.68	0.61
FPG _(mg.dl)_	105.3 ± 22.2	105.3 ± 20.9	105.3 ± 21.2	0.99	0.98	105.4 ± 20.9	104.3 ± 19.6	104.2 ± 23.4	0.77	0.73
Uric acid _(mg.dl)_	4.6 ± 1.2	4.7 ± 1.2	4.6 ± 1.4	0.92	0.86	4.7 ± 1.2	4.7 ± 1.4	4.6 ± 1.2	0.45	0.3
Insulin serum_(µU.ml)_	12.4 ± 11.4	14.4 ± 12.5	14.7 ± 13.6	0.13	0.07	14.4 ± 11.2	13.4 ± 11.9	13.7 ± 12.7	0.32	0.27
HOMA-IR	3.5 ± 3.3	3.7 ± 4.3	3.8 ± 3.3	0.11	0.08	3.9 ± 3.3	3.5 ± 3.4	3.4 ± 3.4	0.31	0.26
HOMA-IS	2.73 ± 2.34	2.59 ± 2.33	2.43 ± 2.06	0.05	0.04*	2.61 ± 2.25	2.71 ± 2.22	2.66 ± 2.28	0.65	0.55
CRP (µg.dl)	0.17 ± 0.16	0.2 ± 0.18	0.22 ± 0.21	0.06	0.04*	0.2 ± 0.18	0.2 ± 0.19	0.19 ± 0.19	0.89	0.87
TyG- index	4.7 ± 2.2	4.8 ± 3.5	5.01 ± 2.6	0.82	0.70	5.3 ± 2.6	4.9 ± 3.6	4.8 ± 2.6	0.55	0.42

*W.DP* Western.dietary pattern, *COPL.DP* Cereals, oil, poultry and legume. dietary pattern, *T* tertiles. *BMI* body mass index, *WC* waist circumference, *FPG* fasting plasma glucose, *HOMA-IR* homeostatic model assessment for insulin resistance, *HOMA-IS* homeostatic model assessment for insulin sensitivity, *CRP* C- reactive protein, *TyG* triglyceride-glucose.

Values are mean ± SD.

MANOVA, analysis of covariance used in crude and adjusted model, and it adjusted for age, sex, education, physical activity, smoking, income and body mass index.

## Discussion

In the present cross-sectional study, we used PCA to derive meal-specific dietary patterns and then, investigated how well values of CVD risk factors and insulin indices change along with the increase in adherence to meal-specific dietary patterns. The analyses suggested a significant association between dietary patterns at breakfast meal and some CVD risk factors, in ways that greater adherence to the “bread, vegetables, and cheese” dietary pattern was associated with a lower FPG and in contrast, adherence to the “oil, egg, and cereals” dietary pattern was associated to a higher FPG concentration, BMI, and TyG index. There were also some suggestions of a positive association between adherence to the “Western” dietary pattern at lunch and dinner meals and BMI and WC and an inverse association with insulin sensitivity, as assessed by HOMA-IS. We also found that adherence to the “bread, cereals, and oil” dietary pattern as afternoon snack was associated to a lower WC. The characteristics of the "Western" dietary pattern at lunch and dinner meals in our study were similar to the Western-style dietary patterns, derived from habitual or meal-specific dietary intake, in other studies conducted in Iran^35,53–55^, Germany^36^ and Brazil^56,57^. A high factor loading of sugar and sweet drinks, processed meat and butter found in the Western pattern was similar to those found in previous studies among Iranian adults^35,53,58^. At breakfast meal, the “bread, vegetable and cheese” dietary pattern identified in our study was similar to a dietary pattern labeled “Mediterranean” found at a breakfast meal in a cross-sectional study of European adolescents^59^, as well as the “Brazilian southeastern” dietary pattern found at a breakfast meal in a large cross-sectional study in Brazil^60^. Our “oil, egg and cereals” dietary pattern at breakfast meal shared similarities with those found in previous studies among European adolescents^59^ and Brazilian adults^61^. In addition, our “cereals, oil, poultry and, legume” pattern found at dinner meal shared similar food groups to dietary patterns labeled “other grains and fat”^62^, “cereals and legumes”^36^, “traditional”^63^, “healthy”^35^, “fruits and vegetables”^61^, “balanced”^64^ found at dinner meals in previous research. However, dietary patterns could be different because of differences in sex^55^, social and economic status, ethnicity^65^, marriage status, and food security^66^ across diverse populations. Also, dissimilarity in beliefs, religions, cultures, educational status and employment status could lead to dissimilarity in dietary patterns ^15,67^.

The association of habitual dietary patterns with cardiometabolic risk factors and obesity has been well established in several studies across different countries^15,68–72^. Totally, these studies showed that a “healthy” dietary pattern that is rich in fish, poultry, nuts, legume, vegetables and fruits is negatively associated with cardiometabolic risk including insulin resistance and inflammation^15,68,73^, whereas a “unhealthy” dietary pattern that is rich in foods such as red meat, processed food, and fried food is directly associated with cardiometabolic risk^15,70^. Previous studies also showed that the “Western” dietary pattern as a unhealthy pattern, is associated with higher insulin resistance^15,70^, metabolic syndrome^15^ and inflammation^74,75^. In contrast, a “Healthy” dietary pattern high in low-fat dairy products, fruit, whole grains, poultry, fish and vegetables was associated with greater insulin sensitivity and lower systemic inflammation^68^. Higher adherence to healthy food patterns such as the "Mediterranean" pattern, with high consumption of olive oil, fruits, vegetables, legumes, and low-fat dairy products was significantly associated with lower CRP^75^. Saghafi-Asl et al. have previously demonstrated that greater adherence to “Traditional” dietary pattern characterized by high intake of fruits and green vegetable, other vegetable poultry, organ meat, red meat and hydrogenated fat was inversely related to lower HOMA-IR^76^. Amini and et al. reported that higher adherence of “Healthy” dietary pattern by higher factor loading of legume, poultry, vegetable, fruits, egg, fish and nut related to lower LAP but not significantly related to TyG index^73^. The same as habitual intake, there are evidence that meal patterns play an important role in the development of cardiometaolic risk factors. A previous study has shown that snacking could alter postprandial glucose, insulin concentrations and satiety^77^. Additionally, eating at the right or wrong time, the duration of calorie restriction, the time of meals and the time of macronutrient intake during a day and even on different days can play an important role in the amount of daily calorie intake and regulating the body weight and fat mass^78^ that are responsible for both insulin resistance and chronic low-grade systemic inflammation^79^. Eating breakfast and consuming fruits and vegetables at breakfast had also a significant relationship with less daily calorie intake^80^. Moreover, the time of meals intake is considered to be an effective factor in health and metabolic function, so that, late dinner increases the risk of obesity/overweight and inflammatory biomarkers^20^.

In the present study, the pattern of “oil, egg and cereals” at breakfast meal had a significant positive relationship with BMI, FPG and TyG-index. In contrast, a higher adherence to the “bread, vegetable and cheese” pattern at breakfast meal was related to a lower BMI. In line with our findings, the pattern labeled "breakfast" among European girls and boys, characterized by high consumption of bread, fruit, cheese and dairy products, indicated an inverse relationship with obesity after controlling for confounders. However, contrary to our findings, the "Mediterranean" and "plant-based and eggs" dietary patterns at breakfast meal did not show a significant association with obesity^59^. Similar to our findings, the “processed-food” dietary pattern in German adults showed a positive association with BMI and WC but not with glycated hemoglobin^81^.

In a recent study, a higher adherence to the "Western" pattern at lunch and dinner was related to a higher WC and BMI and lower HOMA-IS. Also, higher adherence to the "Western" dietary pattern at dinner associated with higher CRP. A positive association between "Western" habitual dietary pattern and obesity was similar to our previous findings^69,82^, although some studies did not find a significant relationship at dinner pattern^17^, in the lunch^63^ and daily pattern^83^. Similar to our findings, previous studies indicated “Western” dietary patterns associated with higher insulin resistance^15,70^ and inflammation^74^.

## Strengths and limitations

We used 24-h dietary recalls, a short-term dietary assessment method, that includes more detailed information about types and amounts of food than long-term assessment methods. All self- reported dietary assessment methods have measurement error, but 24-h dietary recalls are a better measure than FFQ and also, different to FFQ, allow for meal-specific analysis^84^. Most previous studies assessed the association between habitual dietary pattern and disease. According to our knowledge, this is the first study that investigated the relationship between meal-specific dietary pattern and insulin resistance indices. However, under or over reporting of dietary intake is a serious problem related to self-reported dietary assessment methods, particularly in a population with overweight or obese^84^. In addition, subjective decision listed of food groups in PCA, the number of factors extracted and the definition of eating occasions might have a kind of inconsistency in results. Moreover, this is a cross-sectional study which is not able to determine cause and effect relationships. Then, longitudinal study design is recommended to better understanding of causality.

## Conclusion

These results provide evidence that major meal-specific dietary patterns were associated with insulin resistance biomarkers in a sample of Iranian adults. These findings may help inform designing dietary interventions for improving dysmetabolic risk factors. Further prospective studies are required to confirm such relationships.

## Supplementary Information

Supplementary Information.

## Data Availability

The datasets generated and analyzed in current study are available from the corresponding author (SSb) upon request with reasonable justification. The data are not publicly available because they contain confidential information that may compromise the privacy/consent of the participants.
