# Supplementary material for: Meal-specific dietary patterns and biomarkers of insulin resistance in a sample of Iranian adults: a cross-sectional study
Source: Sci Rep. 2023 May 8;13:7423. doi: 10.1038/s41598-023-34235-3 (PMC10167306; doi:10.1038/s41598-023-34235-3)

| Supplementary Table and Figures  Table S1: List of 26 food groups used throughout the analyses | | |  |
| --- | --- | --- | --- |
| Food group code | Name |  |  |
| 1 | Breads | White bread, whole grain bread |  |
| 2 | Cereals & Grains | All pasta and rice, wheat flour, dough, yeast dough, oat, wheat |  |
| 3 | Fresh Fruits & Dried Fruits | All fruit and fresh fruit juices, All dried fruits |  |
| 4 | Vegetables | Leafy green vegetables, Broccoli, cauliflower, cabbage, sauerkraut, Mushrooms, peas, corn, sprouts, garlic, onions, fennel, leek, celery, asparagus, bamboo shoots, vegetable mixes |  |
| 5 | Potato | Potatoes and sweet potatoes |  |
| 6 | Red Meat & Organ Meat | Non-processed meats from: beef, veal, lamb, other red meat, Liver, heart, kidney of cow, goat, lambs and chicken |  |
| 7 | Poultry | Non-processed meats from: chicken, turkey, duck, goose |  |
| 8 | Fish | All fish and shellfish |  |
| 9 | Processed Meat | Sausages, cured ham, salami |  |
| 10 | Broth | Broth |  |
| 11 | Egg | Whole egg, egg whites, yolks |  |
| 12 | Legume | White beans, kidney beans, black beans, other beans, chick peas, lentils |  |
| 13 | Nut | All nuts and seeds |  |
| 14 | Cheese | All cheeses |  |
| 15 | Milk & Dairy Products | Low and high fat milk, dairy beverages, yogurt |  |
| 16 | Liquid Vegetable Oils | All vegetable oils, Olive oil, olive |  |
| 17 | Butter | Butter, Animal oil, hydrogenated oil |  |
| 18 | Pickle | All kind of pickles |  |
| 19 | Salty Snacks | Cracker, chips, cheese puff, corn puff |  |
| 20 | Sugar & Sweets | Honey, syrups, sugar, jams, chocolate, chocolate bars and candies, caramelized fruits and nuts, marzipan, licorice candy, Cakes, cookies, pancakes and waffles |  |
| 21 | Industrial Beverages & Juices | Carbonated and non-carbonated soft drinks, industrial fruit juice |  |
| 22 | Tea & Herbal Tea | Black tea |  |
| 23 | Coffee | Coffee and coffee substitute drinks |  |
| 24 | Sauces | Tomato-based sauces, dips and dressings, mayonnaise-based sauces, dessert sauces, other sauces |  |
| 25 | Spices | Curcumin, cinnamon, pepper |  |
| 26 | Condiments | Vinegar, mustard, herbs, salt, pepper, artificial sweetener |  |

^2^ Number of participants consumed the meals (main meals and afternoon Snack) on all recalled days.

**Figure S1:** **Day screen plot for the principal component analysis**


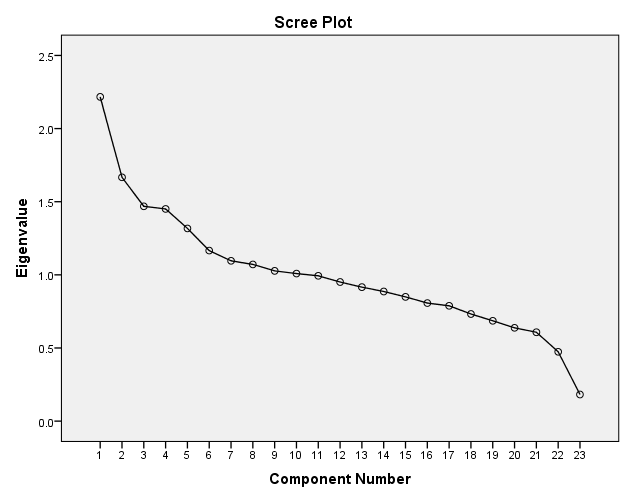


**Figure S2: Breakfast screen plot for the principal component analysis.**


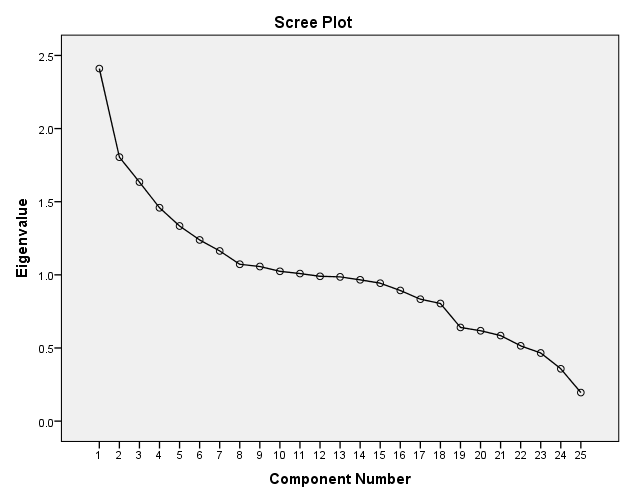


**Figure S3: Lunch screen plot for the principal component analysis**.


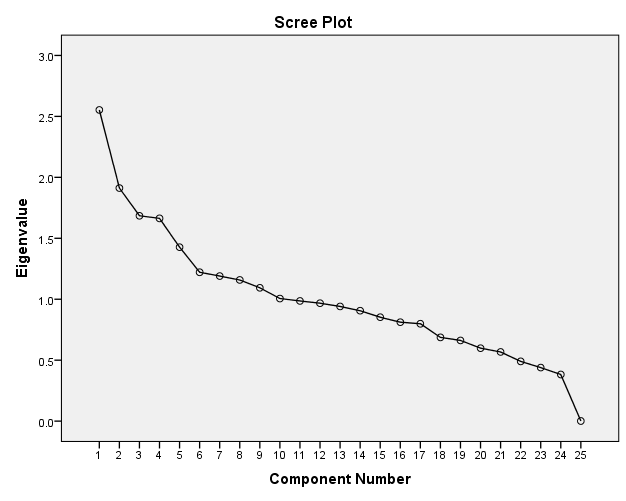


**Figure S4: Afternoon screen plot for the principal component analysis.**


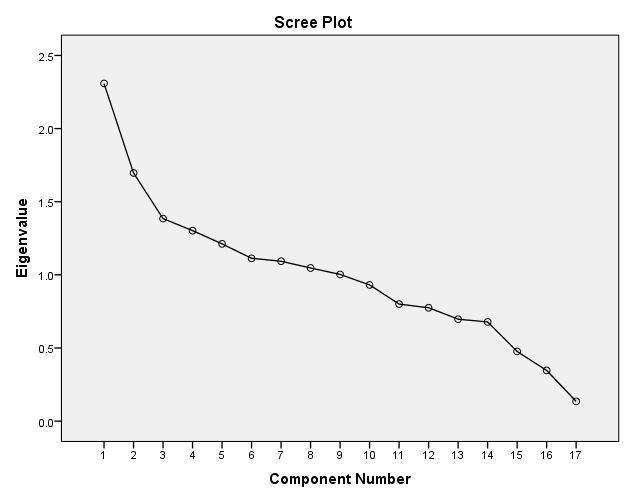


**Figure S5:** **Dinner screen plot for the principal component analysis.**


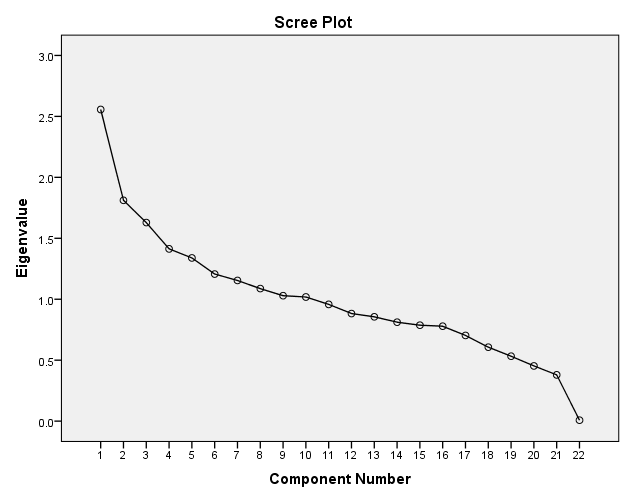

Supplement: Supplementary file 1 — Supplementary Information. [file 41598_2023_34235_MOESM1_ESM.docx]
